# When another assessment attempt is bad for progress

**DOI:** 10.15694/mep.2018.0000147.1

**Published:** 2018-07-19

**Authors:** Steven Burr, Jane May Morrison, Vehid Max Salih

**Affiliations:** 1 Peninsula Medical School; 2 Peninsula Dental School

**Keywords:** resit, retake, grade repetition, summative, assessment, standard

## Abstract

This article was migrated. The article was marked as recommended.

The history and current practice of resit tests are briefly reviewed. The evidence supporting resits and also the problems associated with resits are evaluated. In addition, the financial implications of resits for both students and institutions are explored, along with the need to ensure assessments establish currency of ability and are a reflection of the long-term capability of students. Although resit outcomes are typically capped at the passing score we argue that they still afford an unfair advantage. We conclude that where resit opportunities are provided then they should have higher pass marks than first sit attempts. However, we recommend that the stress of high stakes exams, and thus resits, should be avoided. Consequently, a case is made for an alternative to resits whereby multiple lower stakes assessment results are aggregated.

## Introduction – Background history and current practice

A resit is another attempt at a failed summative examination. The first standardised tests based on merit were the Chinese imperial examinations, introduced in 605 AD, the system continued until 1905 ( Wang, 2013). These examinations could be taken once every three years, with unlimited attempts. Successful candidates were ranked according to merit and awarded corresponding positions as officials of state. Later the first universities were established in Europe, starting with Bologna in Italy in 1088 ( Sanz and Bergan, 2006). Universities adopted the tradition from the Roman and Greek education systems in using disputation for assessment ( Wilbrink, 1997). This continued until students began to be ranked for progression based on merit by Cele (1375-1425) in the Netherlands ( Wilbrink, 1997). In 1702, Trinity College Cambridge introduced the first shift in Europe from oral to written examinations ( Ball, 1889). Advancement through patronage began to decline in Europe following the founding in 1806 of the East India Company College, which was directly modelled on the original Chinese imperial examination system. This then provided the basis for the British Civil Service Commission in 1855, whereby competitive examination for entry required candidates to be between 21-24 years of age, which made three attempts permissible ( ARC, 2008). It is clear that repeat attempts have been available since the inception of examinations, but for the first 1250 years repeat attempts were only available in the next examination cycle. Since the British introduced limits on the number of attempts (by age) the provision for repeat attempts has diversified. This applies universally to degree programmes, including medicine and allied professional healthcare subjects.

The current practice at the World’s top 30 universities ( Times Higher, 2018) can be readily determined from their individual websites. Of these, 20 universities are in the USA where course repeats occur. Universally in the USA failed courses appear on the student’s permanent record and transcript. Failure may be compensated by taking an extra course, or by repetition. Practice varies between these institutions as to whether repetition: requires approval; is only be permitted if below a certain grade; is permitted to count for credit and towards the Grade Point Average (and then whether only the first repeat counts, or all repeats); and whether repetition is limited to one, two or three repeat attempts. At the Swiss Federal Institute of Technology in Zurich students are required to repeat courses they have failed in the following academic year. In contrast to the modern trend, prior to 1983 students at the University of Washington were able to request unlimited repeats with only the final repeat counting to their Grade Point Average.

The UK universities in the top 30 all permit one resit at the end of the same year or in the following academic year, either with or without attendance. Typically, resits are only permitted if required for progression and the outcome is typically capped at the pass mark. One exception is the Tripos examination taken at Cambridge University which permits no resit, still stipulating that ‘no student who has been a candidate for any Part shall again be a candidate for the same Part’. In Canada and Singapore the systems are similar to that generally found in the UK. At Ludwig Maximilian University of Munich no more than two retakes of any examination are permitted. Interestingly, Peking University also permits only two retakes, but the grade of the first retake is taken as the final grade, with the second retake grade capped at 60% and if the second retake is failed then this stands as the final grade. At the Karolinska Institute up to six resit attempts are permitted. In contrast to the variation within nations elsewhere, Austria has a national standard whereby all university students are entitled to repeat failed exams three times within an unlimited timescale provided registration is continuous.

It follows that in the UK most universities now consider the provision of resits to be a fundamental part of their assessment systems, but how resits are implemented does vary. The fairness of providing resits presupposes two conditions: Firstly, that the standard of a resit is equivalent to that of a first sitting. Secondly, that the intervening time presents the opportunity to rectify sufficient deficiencies, i.e. remediation, in order to achieve the required standard. The main problem is that there is very little theory and even less empirical evidence to explain or support the provision of resit examinations as opposed to repeating a course. Resits are a relatively unexplored area of the assessment process that is clearly crucial to student degree outcomes. We aim to seek to fill this gap with a conceptual framework for consideration of the factors affecting resits and factors affected by resits. Given the current paucity of empirical evidence our more specific objective in this paper is to evaluate the rationale and evidence for offering examination resit opportunities to undergraduate students. Ensuring that resit opportunities within medicine and applied professional healthcare programmes are fit for purpose clearly has patient safety implications.

## Evidence supporting resits

The literature on resit and supplementary examinations was considered scarce when it was reviewed by Ricketts in 2010 ( Ricketts, 2010), and it remains so six years later. Searching the literature using the Education Resources Information Center ( www.eric.ed.gov) reveals 11 peer-reviewed publications for ‘resit’, 55 for ‘retake’ and 478 for ‘grade repetition’. In comparison there are 1,930 peer-reviewed publications for ‘summative’ and 65,764 for ‘assessment’. Given the paucity of data available on resits, we propose a conceptual structure to evaluate the use of resits based on a review of the available literature. There are practices and assumptions, but little supporting theory or analytical studies. Common practices (as indicated above from the top 30 universities) include: not permitting those who were successful at their first attempt to take a resit; limits on the number of resit attempts; and, that students sitting resit exams have their results capped and thus should only be allowed the equivalent of a ‘pass’ and no higher. Possible assumptions include: that students sitting resits have an unfair advantage over other students because they have an additional opportunity to improve; that there is an undesirable ‘resit culture’ amongst certain students who are otherwise disengaged from their programme and rely on resits to pass; and, that resits still need to be offered despite their disadvantages. There is published evidence suggesting that: candidate ability increases with up to three attempts, but ability decreases thereafter ( McManus, 1992); repeating a class increases attainment due to increased ‘learning effort’ ( Fertig, 2004); and shortening the interval between first sit and resit increases candidate performance and the rational use of time by all concerned ( Slater, 2009). Less certain is the longevity of any increase in performance associated with resits. It has been shown that candidates who pass at resit do perform better in the following stage of the course compared to those who passed at the first attempt ( Proud, 2015), although others have shown that this increase in performance is not always significant ( Pell *et al*., 2012; Arnold, 2016). This suggests that the effect can be transient for some candidates or under some conditions, and thus may be influenced by other factors such as variable effectiveness of remediation in feeding forwards, and temporary increases in motivation or attention.

It is important to understand the rationale and purpose of a resit assessment. Resits may be provided to reduce the psychological stress of first attempts, reduce the financial and reputational costs of failure, and ensure that students can go on to maximally fulfil their potential contributions to society. However, we postulate that there is an overriding duty to maintain academic standards. Hays (2008) notes that resits should not be used to allow student learners with serious deficiencies to learn superficially and obtain a lucky pass, but rather to see if students with borderline scores achieve a similar score in two examinations. If performance improves to more than one standard error above the pass mark, then a pass decision is supported, while the same or a lower score supports a fail decision. Thus the role of assessors is to assess the resit candidates against the exact same standards as in the original assessment, so that it is then possible to judge whether performance has improved sufficiently.

## Problems with resits

The rationale for offering a resit is to provide an opportunity to attempt a test again without prejudice due to the outcome of a first attempt. Thus the resit must be set at the same standard as the first test, although the equivalence of both difficulty and quality can easily be challenged by logic.

The additional intervening time following a first sitting places the resit further from the relevant learning opportunity and makes the test harder; while providing more time to prepare for the test potentially makes the test easier. Having taken the test before lowers the cognitive load, effectively making the test easier. Teaching staff may also give cues to students as to what to expect. Furthermore, assessors with awareness that they are marking a resit may be biased when applying their academic judgement. Some assessors may be more lenient knowing that the assessment has a high stake value to the candidate, whereas other assessors may be harsher knowing that the assessment has been previously failed. The extent to which these various factors influence the difficulty of the resit is likely to vary with each specific circumstance. Furthermore, any resit will typically be taken by a minority of students. A smaller number of students reduces the probability of flagging potential question errors, and precludes reliable psychometric analyses of facility and discrimination. Thus less data is available for post-test decisions on the potential removal of questions from resit tests and consequently resit tests are at risk of being of lower quality.

Both capping of resit outcomes and the timing of resit provision can also be considered unfair. When a test result needs to be carried forwards for some form of ranking or classification purposes, then using either the first or resit result can be considered unfair. If first sit marks are used then, when taking a resit, there is less incentive as the possibility of improving the final outcome from the programme has been removed. However, if resit marks are used then students could adopt a strategy which spreads the assessment load between first sits and resits. Thus resits are often capped to prevent those who passed first time with low scores from being unduly disadvantaged.

Within year resit opportunities present yet further concerns. A student must be deemed capable of coping with the next stage before being permitted to progress. A resit would require the preparation of an additional test in case anyone has a validated extenuating/mitigating circumstance for the resit. Importantly, the scheduling of an additional test following a resit could be problematic. Depending on the nature of the extenuating circumstance, it runs a real risk of overlapping into the subsequent academic year. This potentially unfairly reduces the student’s opportunity to engage with the next stage of the programme. The impact is two-fold: the student will need to divert time to prepare for and take the additional test; and, perhaps more importantly, study the next stage without having demonstrated capability for it. In contrast, requiring a complete year to be reattempted would ensure comparable standards and currency of capability with advancement through the programme, but is associated with a considerable additional financial cost to the student.

## Financial costs should focus on students

The extent of the cost to the student, institution, profession and society depends on the stage at which a student fails. A resit opportunity before the start of the next academic year enables some students who fail at the end of a year to improve sufficiently to progress into the next year. If a student is lost from a programme then they (and their fees) are lost from that cohort for all remaining years of that programme. Students repeating complete years potentially increase the income from fees. The provision of resits also adds to the reoccurring annual workload, with the associated increase in resource costs. Resits can only benefit those who take them, and providing resits requires the diversion of resources from other parts of the curriculum unless resit fees are charged. Thus there is a financial disincentive for universities to remove students early. This is in conflict with the best interests of students, who benefit most when academic failure is identified as early as possible; so as not to waste time on a programme when they could be doing something else, nor pay unnecessary fees. The student-centred approach would be to have failure rates minimised by effective recruitment selection, management of expectations, formative opportunities prior to all summative tests, and comprehensive support and rapid remediation processes; coupled with withdrawal rates being highest early in the programme.

## Professions need to establish currency of ability and long-term reflection of capability

A 2003 study by McManus *et al* found that academic achievement predicted certain aspects of successful medical career progression, like the number of research papers published, but there was no evidence to show that it predicted excellence in doctor-patient interaction or clinical care ( McManus *et al*., 2003). Another study found that exam scores were not a good predictor of evaluations of medical students on a surgery clerkship ( Goldstein, 2014). Similarly, the effect may hold true between stages of education; Powlis *et al* (2007) found that prior academic achievement accounted for only a small amount of success at medical school, and that students who had resat their university entry examinations did not, as a group, go on to experience failure at medical school.

Nevertheless, students are expected to develop their skills at a particular rate; learning eventually is not as positive as learning within a designated time-frame ( Pell *et al*., 2009). The ability to absorb new information relatively quickly and at a deeper level is arguably a desirable trait, particularly in courses that require registration with a professional regulatory body. Depending on the subject area, a student repeatedly demonstrating inability to do this might be problematic. This concern over the salience of time limits was the apparent rationale behind changing the rules on nursing Common Foundation Practice exams ( Waters, 2006).

Students who pass at resit typically perform better in the following stage than those who pass at the first attempt ( Proud, 2015). It might be expected that a student resitting an exam in August has an academic advantage over students who pass first time and don’t study over the long summer break. A student who has successfully completed resit examinations presumably has the previous year’s learning ‘fresh in their mind’ when term begins. However, the work taught in second year might bear no relation to the work taught in first year. The work may not build on the same concepts or use the same techniques. So if a student does improve as a result of having to do a resit, it might be because their near-failure motivated them towards a change of attitude, not because their overall knowledge or skill level improved ( Proud, 2015).

## Resits can afford an unfair advantage due to luck

In a poor assessment setting, the more times an examination is taken the more likely that a successful outcome will occur by chance. For example, this is true where questions are reused from a bank and there is the possibility that the same candidates will see similar questions again. The more times an examination is taken the greater the probability of questions being presented which have been seen by that candidate before. The element of pure luck becomes especially problematic in cases where high numbers of resits are being taken. Waters (2006) recorded as many as five attempts at a first-year exam, whilst another study found that (since the Royal College of Physicians of the United Kingdom placed no limit on the number of attempts) in the most extreme cases postgraduate candidates had sat Part 1, Part 2 and Clinical examinations 26, 21 and 14 times respectively ( McManus and Ludka, 2012). However, high numbers of attempts are by no means the norm, especially for undergraduate exams. It is clear that more than one resit attempt is discouraged and considered rare at most universities (as indicated above from the top 30 universities). The reasons for this may vary and possibly include: the institutional policy on charging resit fees; the availability of administrative resources for implementing resit assessments; and, insufficient time to process the progression of a successful resit candidate from one academic year to the next.

Regardless of these common practices, some groups working in education theory believe that the advantages of allowing students multiple resits are surprisingly high. McManus and Ludka analysed candidates’ grades using multilevel modelling, fitting negative exponential growth curves to individual candidate performance. They modelled longitudinal performance across three sittings and found evidence of improvement. Candidates continued to show evidence of true improvement up to at least the tenth attempt at their MRCP(UK) Part 1 exam ( McManus and Ludka, 2012). The authors acknowledge the problematic role of luck (as described above) in these candidates’ passing, and ultimately find it difficult to justify allowing candidates to pass by sheer dint of probability. However, they do not rule out offering multiple resit chances, and tentatively recommend finding ways to increase the pass grade for each successive attempt ( McManus and Ludka, 2012). Proud (2015) comes to a similar conclusion, that a first-year resit exam might include a higher threshold for progression into second year, after finding that microeconomics students who performed well in a resit examination outperformed both their peers who performed badly in the resit and their peers who did no resit at all. Proud (2015) adds that this might disadvantage students who currently only marginally pass, but as Ricketts (2010) points out, it has to be decided which is worse: a false positive (students succeeding where they should have failed); or, a false negative (students failing where they should have succeeded). This issue is clearly a concern in professional subjects where graduates become responsible for the safety of others.

## If there must be resits then they should have higher pass marks

Pell *et al* found that students who resat their exams did appear to have a statistical advantage over other students ( Pell *et al*., 2009). This provides tentative numerical confirmation of the common assumption, as stated by Ricketts: “Staff felt that resits were somehow easier than taking the exam the first time” ( Ricketts, 2010). Using two datasets taken from two medical schools’ exam scores, Pell *et al* analysed the distribution of students’ grades, noting that the pass mark is usually set at two standard deviations below the mean and consistently fails about 3% of a cohort. They found that the mean of each medical school’s resit marks was comparable to the mean of the marks for students who passed first-time, but logically it shouldn’t be. The resit students were a biased sample who had already demonstrated lower-level academic abilities, and yet when the total failed exams were aggregated, 5.9% failed the first assessment and 6.6% of that 5.9% failed the resit, not a statistically significant difference ( Pell *et al*., 2009). Pell *et al* therefore recommended setting the pass marks for resit at a higher level than the first sit exam. This would undoubtedly make the experience of resitting students more stressful, but might be considered fairer to those students who passed first time.

## The stress of high stakes exams should be avoided

As an extreme example, one survey has found approximately 30% of those medical students polled were suffering from a mental illness, and in some cases this was so severe that 15% reported suicidal thoughts ( Billingsley, 2015). Just over half of those polled also reported generally very high levels of anxiety. Other studies have found medical students appear to be at an elevated risk of depression and suicide, particularly during high stakes assessment episodes ( Kamski *et al*., 2012). The high anxiety of sitting an assessment, affects not only the students’ wellbeing, but also affects their ability to perform well in the assessment ( Encandela, 2014). An older study of overseas students, studying medicine at Edinburgh University, found that symptoms of anxiety, depression and headaches in February correlated strongly with first year exam failure in May ( Cox *et al*., 1981). This is a particular problem, as it appears that many mental health disorders impair cognitive function in key domains needed to succeed in academic assessments, such as visuospatial processing and language domains ( Baune *et al*., 2009). Overall, the published evidence implies that examinations are stress-inducing and contribute to mental illness and, by extension, resits are more dangerous because they are higher stakes. This then provides some justification for lower-stress assessment methods that do not place excessive weight on one key moment of academic performance.

## Discussion - The case for an alternative to resits and repeats

As resits have deviated from the traditional model of repeating in the next session, the tendency has been to facilitate an additional opportunity to progress by including a resit examination within the same session. This may be more attractive to students than repeating a year, but a second chance mentality is not the best preparation for life. There is mounting evidence that over the last decade there has been a failure to fail students, particularly in the healthcare professions ( Yepes-Rios *et al*., 2016). The natural inference is to remove resits and return to complete repeats in subsequent sessions. However, an alternate approach is possible based on accumulating evidence about a student’s performance.

The primary purpose of a resit should be to obtain more information on student performance so as to increase the statistical power of a decision ( Ricketts, 2010). This could be achieved by removing resits and introducing sequential testing, with longer tests for weaker students and shorter tests for stronger students ( Pell *et al*., 2013; Wainer and Freinburg, 2015). Alternatively, resits could be graded in a different way ( Ricketts, 2010). On the principle that more data always gives better information about a circumstance, Ricketts argues that it is wrong to simply ‘cancel out’ a failed exam score with a new resit score as if the first exam had not taken place. Instead, he argues in favour of adding together both scores and taking an average. In recent years this has been successfully adopted at the Peninsula Medical and Dental Schools at the University of Plymouth in the UK. Clinical examinations are split into two phases, each with the same number of stations ( Huline-Dickens *et al*., 2014). Students who pass the first phase are exempt from the second phase. Students who fail to achieve the overall pass mark for the first set of stations are required to return and take a second set 2-4 weeks later; the outcome of which is derived from the average performance across both sets measured against the average pass mark from both sets. As skills should have been practiced throughout the academic year, a lengthy remediation period is not appropriate.

How could providing another chance at knowledge assessments be incorporated in a fairer and timely way to ensure remediation of deficiencies? There are a few examples of courses in the UK, which have an alternative structure to the conventional end of year May exams, plus August/September resits. These are in a definite minority and some do not vary from the conventional model in radical ways. Of these, progress testing is perhaps the most radical. With progress testing, students in all years sit the same test that is set at the final year qualifying standard ( Freeman *et al*., 2010). There are several such tests spaced at regular intervals within each year. Scores are graded, and grades aggregated across tests. Thus tests are frequent and an individual test is low in stakes, reducing stress and promoting continuous learning (in direct contrast to resits). Whilst it is possible that some students may be anxious about more frequent testing, and may even prefer fewer higher stakes tests, there are other benefits. Each student can map his or her individual growth towards the final standard required. Thus, the final goal is transparent from the beginning, with ample opportunity for early remediation ( Ricketts and Bligh, 2011). The evidence in support of not incorporating resits is that progress testing; (1) provides an equivalent to resits with multiple tests and (2) produces lower than average overall failure rates and better than average preparedness for practice, when compared with assessment processes which incorporate resits. These two points are elaborated below:

1. Students are required to sit multiple tests spaced throughout the year, with targeted support and remediation between tests. A degree of compensation across tests effectively provides an equivalent opportunity to resits, but more constructively by focusing on continuous engagement and progress. Additional chances are effectively included within aggregate performances over multiple low stakes assessments that apply equally to all students and so are fairer. To offer a resit after the summer break would allow independent preparation over the summer, but would effectively be offering the equivalent of a third attempt and would not demonstrate engagement, progress, or whether the student is capable of coping with the next academic year of study. Failure to demonstrate capability within an academic year is indicative of insufficient capability to cope with the next stage of study. Thus, for failure of a single module there should be an opportunity to repeat the year to provide evidence that the student has both the aptitude and self-motivation to apply themselves appropriately for the next stage of study. To permit progression after an end-of-summer resit would exacerbate poor engagement, fail to demonstrate progress, and set a student up for failure in the following academic year.
2. Progress testing is associated with a lower than average overall failure rate, so students who fail are statistically more likely to have failed (after resits) had they been studying on a programme which provided resit opportunities ( Norman *et al*., 2010). Programmes with progress testing are also associated with a higher preparedness for practice than other programmes in the same subject ( Bleakley and Brennan, 2011; Ali et al. 2015). The approach to assessment is different with progress testing, but this difference enables more timely support for students who are close to failing and in ensuring that they are appropriately prepared for practice.

The difficulty with progress testing is that the self-directed learning expected is very different to the traditional style of learning and many students find it difficult, particularly during the early stages of courses involving progress testing, to manage their own learning. Many students take time to understand the depth of learning required, as they have never had to do this before. Thus, resits may be appropriate for the first year to facilitate the transition. However, less emphasis on progress testing in the first year may translate into a delayed growth in performance across the course.

## Conclusions

Resits are widely used in many universities and resits may be helping students to progress by indirectly lowering academic standards. Even when the actual resit test is of the same standard as a first sit test, the circumstances surrounding the resit can reasonably be considered to cause a difference in difficulty and quality. In addition, both capping and timing have the potential to be unfair, along with an element of luck in poorly designed resit tests. There may also be adverse long-term implications for student finances, professional learning, and the mental wellbeing of the students.

The diversity of resit provision across the higher education sector undermines the fair assessment of comparable academic standards. If there is an additional opportunity for students who were initially unsuccessful to improve their performance, then the standard required should be higher. This mandates further empirical study to quantify how much higher should pass marks be for resits compared with first sit examinations. However, it would be better for students if additional opportunities were available to all and comprehensively integrated into the curriculum, as with the example of progress testing. Given frequent testing and remediation opportunities, and consequently fewer students failing overall than programmes that do have resits, then those students who do fail a stage in a programme with progress testing can only demonstrate capability by repeating that stage and being successful. This is supported by evidence that suggests the use of progress testing instead of resits leads to graduates who are better prepared for practice.

## Take Home Messages

When has a failing student failed? In summary, a single assessment after a summer vacation cannot demonstrate engagement nor sufficient progress to ensure that a student could cope with the next stage of a programme.

## Notes On Contributors

Dr Steven Ashley Burr is an Associate Professor and Deputy Director of Assessment for Medicine and Dentistry at Peninsula Medical School, University of Plymouth.

Jane May Morrison, was formerly a temporary Research Assistant at Peninsula Schools of Medicine and Dentistry, and is now a PhD student at the University of Exeter.

Dr Vehid Max Salih is an Associate Professor (Reader) and Deputy Director of Assessment for Dentistry and Medicine at Peninsula Dental School, University of Plymouth.
