# Supplementary material for: When another assessment attempt is bad for progress
Source: MedEdPublish (2016). 2018 Jul 19;7:147. [Version 1] doi: 10.15694/mep.2018.0000147.1 (PMC10701820; doi:10.15694/mep.2018.0000147.1)
Supplement: Peer review history

# Reviewer report: Reviewer response for version 1

**DOI:** 10.21956/mep.19625.r29205

**Published:** 2018-07-26

**Authors:** Ian Wilson

**Affiliations:** 1 International Medical University

This review has been migrated. The reviewer awarded 4 stars out of 5 I found this an interesting and challenging paper. It takes the very limited literature on resits and extrapolates them to a more extensive practical review of the process of managing students who fail an examination.This is something that challenges most medical schools. Schools want to do the best for their students, but they also are cognisant of the need to ensure that those who progress through the medical course will be competent and safe practitioners.the paper is a very sound introduction to the topic, but the recommendations seem to be not based on the literature. Whatever decisions are made concerning student who fail are arbitrary and reflect more the philosophy of the school than being soundly based on the literature.This paper is a good paper to stimulate discussion of an important topic

Reviewer Expertise:

NA

No decision status is available

# Reviewer report: Reviewer response for version 1

**DOI:** 10.21956/mep.19625.r29206

**Published:** 2018-07-26

**Authors:** Michael SH Wan

**Affiliations:** 1 University of Notre Dame

This review has been migrated. The reviewer awarded 4 stars out of 5 An interesting article reviewing the pros and cons of a resit (supplementary) exam. Thorough review of the literature on this topic.In our medical school (in Sydney), we offer resit only for the final year students. In the past 10 years, we only have students requiring to resit the OSCE when their scores were below the pass mark set by borderline line regression in the OSCE or if they failed more than a pre-set number of stations AND their score was within 1 SEM of the pass mark (when they are in the borderline zone) where we could not make a clear P/F judgement. The resit OSCE obviously required a significant additional resources to set up and additional examiners/simulated patients. The students would have 2-3 weeks to "catch-up" with their clinical skills before resit. For the standard of the resit OSCE, we re-used a mixture of previous years summative OSCEs where we had clear pass marks (using borderline regression on the whole year cohort) selected from a pool of more than 80+ cases. We did not increase the pass mark for the resit. In our experiences, many studeents who were given a resit just had a "bad day' in the actual summative OSCE and they performed well above the pass standard in the resit. A few of them failed the resit OSCE, repeating the whole year; and then performed much better after repeating.We had no students failing the end of final year written exams in the various domains. I agree that progress testing and more within year continuous assessment (multiple assessment points) will certainly reduce the stress for the end of year summative exam and also allow early identification of struggling students for proper feedback and remediation. The programmatic assessment model is certainly a viable option for better assessment and feedback for progression.

Reviewer Expertise:

NA

No decision status is available

# Reviewer report: Reviewer response for version 1

**DOI:** 10.21956/mep.19625.r29203

**Published:** 2018-07-19

**Authors:** P Ravi Shankar

**Affiliations:** 1 American International Medical University

This review has been migrated. The reviewer awarded 4 stars out of 5 This is an interesting paper which explores various aspects of ‘resit’ exams. Resit or repeat exams were not common when I went to medical school. If you failed an exam you had to repeat the course and take the exam again after six months with the next batch of students. The only occasion on which we got a resit was during the final year exam in which many students failed due to various reasons. Many passed during the resit exam held a month after the results of the first exam were declared. Our faculty members, however, were of the opinion that students had used ‘shortcuts’ and were not favorably disposed toward students who had passed during the resit. The situation may be at the other extreme in offshore Caribbean medical schools. Many of these schools admit students three times a year, in January, May and September. Students who fail their exams during the basic sciences get another opportunity to pass the exams after three weeks. Many of the issues mentioned by the authors are true in the Caribbean setting also. Caribbean schools do not want students to transfer to other schools in expectation of a better academic performance or results in various exams. It is easy to transfer between schools and competition among schools is fierce. The authors have discussed the issue of resit exams in detail in this manuscript. I was not previously aware about progress testing. The history of examinations has been well presented. The paper is well-written and addresses a subject which is of importance in medical education but is not often studied or researched.

Reviewer Expertise:

NA

No decision status is available

# Reviewer report: Reviewer response for version 1

**DOI:** 10.21956/mep.19625.r29204

**Published:** 2018-07-19

**Authors:** Alex Moroz

**Affiliations:** 1 New York University School of Medicine

This review has been migrated. The reviewer awarded 4 stars out of 5 There is some evidence in the medical specialty board examinations in US (which have a time limit for passing and becoming 'board certified') that for students who fail the initial examination, the chances of passing decline sharply and progressively on subsequent attempts. So I am not sure about the 'chance' unfairness. (Robinson LR, Sabharwal S, Driscoll S, Raddatz M, Chiodo AE. (2016) How Do Candidates Perform When Repeating the American Board of Physical Medicine and Rehabilitation Certification Examinations? Am J Phys Med Rehabil, 95(10):718-724.)I think your suggestion of multiple smaller, lower stakes assessments throughout the year that are used together for summative decisions fits very well with Schuwirth and van der Vleuten's notion of a system of assessment. (Lambert W. T. Schuwirth & Cees P. M. Van der Vleuten (2011) Programmatic assessment: From assessment of learning to assessment for learning, Medical Teacher, 33:6, 478-485)

Reviewer Expertise:

NA

No decision status is available
